# Tooth Loss and Carotid Intima-Media Thickness in Relation to Functional Atherosclerosis: A Cross-Sectional Study

**DOI:** 10.3390/jcm11143993

**Published:** 2022-07-10

**Authors:** Yuji Shimizu, Hirotomo Yamanashi, Masayasu Kitamura, Jun Miyata, Fumiaki Nonaka, Seiko Nakamichi, Toshiyuki Saito, Yasuhiro Nagata, Takahiro Maeda

**Affiliations:** 1 Department of General Medicine, Nagasaki University Graduate School of Biomedical Sciences, Nagasaki 852-8501, Japan; yamanashi@nagasaki-u.ac.jp (H.Y.); seiko-n@nagasaki-u.ac.jp (S.N.); tmaeda@nagasaki-u.ac.jp (T.M.); 2 Department of Cardiovascular Disease Prevention, Osaka Center for Cancer and Cardiovascular Diseases Prevention, Osaka 536-0025, Japan; 3 Leading Medical Research Core Unit, Nagasaki University Graduate School of Biomedical Sciences, Nagasaki 852-8523, Japan; syto@nagasaki-u.ac.jp (T.S.); ynagata1961@nagasaki-u.ac.jp (Y.N.); 4 Department of Oral Health, Nagasaki University Graduate School of Biomedical Sciences, Nagasaki 852-8588, Japan; masayasu-kitamura@umin.ac.jp; 5 Department of Islands and Community Medicine, Nagasaki University Graduate School of Biomedical Sciences, Nagasaki 853-0031, Japan; j-miyata@nagasaki-u.ac.jp (J.M.); f.nonaka@nagasaki-u.ac.jp (F.N.); 6 Nagasaki University Health Center, Nagasaki 852-8521, Japan; 7 Department of Community Medicine, Nagasaki University Graduate School of Biomedical Sciences, Nagasaki 852-8523, Japan

**Keywords:** arterial stiffness, CAVI, CIMT, functional atherosclerosis, tooth loss, atherosclerosis

## Abstract

Structural arterial stiffness can be evaluated with carotid intima-media thickness (CIMT). Functional arterial stiffness can be evaluated with cardio-ankle vascular index (CAVI). A positive association between CIMT and tooth loss has been reported, but no studies have evaluated the association between CIMT and tooth loss in relation to functional arterial stiffness (functional atherosclerosis). A cross-sectional study of 1235 Japanese individuals aged 40–89 years was conducted. Tooth loss was defined as being in the lowest tertile for the number of remaining teeth (≤20 in men and ≤19 in women). Functional atherosclerosis was defined as CAVI ≥ 9.0. Independent of known confounding factors, CIMT was positively associated with tooth loss only in participants without functional atherosclerosis. Adjusted odds ratios for tooth loss and a 1 standard deviation increment in CIMT were 1.27 (1.04–1.55) for participants without functional atherosclerosis and 0.99 (0.77–1.26) for participants with functional atherosclerosis. CIMT and functional atherosclerosis had a significant effect on tooth loss; the fully adjusted *p*-value for the interaction on tooth loss was 0.019. Independent of known confounding factors, CIMT is positively associated with tooth loss only in participants without functional atherosclerosis. This finding helps clarify the influence of the progression of arterial stiffness on tooth loss because the progression of structural atherosclerosis might have a beneficial influence on the maintenance of the microcirculation.

## 1. Introduction

A significant positive association between carotid intima-media thickness (CIMT) and cardio-ankle vascular index (CAVI) has been reported [1]. However, these two parameters reflect different aspects of arterial stiffness. CIMT evaluates structural arterial stiffness while CAVI evaluates functional arterial stiffness.

Increased arterial stiffness is a well-known pathology of atherosclerosis. Generally, functional arterial stiffness (atherosclerosis) and structural arterial stiffness (atherosclerosis) are considered essentially the same medical condition. Even though, from the perspective of endothelial repair activity, there are significant differences between those types of arterial stiffness (atherosclerosis). Aggressive endothelial repair increases both functional and structural arterial stiffness (atherosclerosis), while insufficient endothelial repair furthers functional arterial stiffness (atherosclerosis) but not structural arterial stiffness (atherosclerosis) [2,3].

Hematopoietic stem cells known as CD34-positive cells play an important role in endothelial repair by differentiating into mature, CD34-negative cells such as endothelial cells [4], macrophages, and foam cells [5]. Since macrophages [6] and foam cells [7] contribute to the development of pathological atherosclerosis, CD34-positive cells are necessary for the development of structural atherosclerosis [8]. Therefore, shortage of CD34-positive cells increases CAVI but not CIMT [9]. Furthermore, due to consumption, aggressive vascular repair that relates to the development of structural atherosclerosis, also causes a reduction in circulating CD34-positive cell count [9]. This is the reason why no significant association between CIMT and circulating CD34-positive cells were observed among participants with sufficient CD34-positive cells [9]. Therefore, the number of circulating CD34-positive cells acts as a significant indicator of endothelial health. However, circulating CD34-positive cells are not easily and routinely measured. Then, we focus on CIMT and CAVI.

A previous Japanese study reported that fewer teeth could be significantly related to atherosclerosis as evaluated with CIMT [10]. Another Japanese study reported a linear relationship between tooth loss and degree of arterial stiffness evaluated with CAVI [11]. Those studies indicate that tooth loss is significantly associated with arterial stiffness evaluated with either CIMT or CAVI.

Since structural changes in atherosclerosis are caused by aggressive endothelial repair while functional atherosclerosis is caused both by aggressive and insufficient endothelial repair [2,3,9], the status of functional atherosclerosis could act as a strong confounding factor on the association between structural arterial stiffness values (CIMT) and tooth loss.

Therefore, we hypothesized that there is a positive association between CIMT and tooth loss only among participants without functional atherosclerosis. To evaluate our hypothesis, a cross-sectional study was conducted.

## 2. Materials and Methods

### 2.1. Study Population

A cross-sectional study of 1235 Japanese individuals aged 40–89 years who participated in an oral health check-up in 2016–2018 was conducted. The study population comprised 2298 participants (833 men and 1465 women) aged 40–89 years from Goto City in western Japan who attend an annual health check-up with an oral health assessment in 2016–2018. This annual check-up program was conducted by the local government under the direction of the Ministry of Health, Labor, and Welfare in Japan. Participants without remaining teeth (*n* = 1060) or without laboratory data (*n* = 1) or CAVI data (*n* = 2) were excluded from the analysis. The remaining participants, 486 men and 749 women, were included in the study. The mean age was 68.5 (standard deviation (SD), 10.5) years for men and 68.0 (SD, 10.1) years for women. This study was approved by the ethics committee of the Nagasaki University Graduate School of Biomedical Sciences (project registration number, 14051404-13). All study procedures performed involving human participants were in accordance with the ethical standards of the ethics committee and the 1964 Helsinki Declaration and its later amendments for comparable ethical standards.

### 2.2. Data Collection and Laboratory Measurement

The data for this study were obtained from annual health check-ups that included an oral health assessment. Trained interviewers obtained information on smoking status. Body weight and height with bare feet and light clothing were measured using an automatic body composition analyzer (BF-220; Tanita, Tokyo, Japan). Body mass index (BMI) was calculated as (weight in kg)/(height in m)^2^. After at least 5 min of rest, blood pressure (systolic and diastolic) was measured in the sitting position using a blood pressure measuring device (HEM-907; Omron, Kyoto, Japan).

Fasting blood samples were collected. Triglycerides (TG), high density lipoprotein cholesterol (HDLc), hemoglobin A1c (HbA1_C_), and γ-glutamyltranspeptidase (γ-GTP) were measured using standard laboratory procedures at SRL, Inc. (Tokyo, Japan).

#### 2.2.1. Measurement of Carotid Intima-Media Thickness (CIMT)

An experienced vascular technician measured CIMT with ultrasonography of the left and right common carotid arteries using a LOGIQ Book XP with a 10-MHz transducer (GE Healthcare, Milwaukee, WI, USA). Mean values for left and right CIMT that did not include plaque measurements were calculated with a semi-automated digital edge-detection software (Intimascope; MediaCross, Tokyo, Japan) and a protocol that has been described in detail elsewhere [12].

#### 2.2.2. Measurement of Cardio-Ankle Vascular Index (CAVI)

Functional atherosclerosis was defined as CAVI ≥ 9.0, as in our previous study [13]. CAVI was determined with a Vasera VS-1000 vascular screening system (Fukuda Denshi, Tokyo, Japan) with the participant resting in a supine position. The underlying principles of CAVI have been described elsewhere by Yambe et al. [14].

Brachial-ankle pulse wave velocity (PWV) is generally used to evaluate arterial stiffness. Since PWV measurements can be strongly affected by blood pressure [15], CAVI was recently developed in Japan to avoid the susceptibility of PWV measurements to blood pressure differences [16].

#### 2.2.3. Oral Examination

Trained dentists performed periodontal examinations using a modification of the method used in the Third National Health and Nutrition Examination Survey [17]. Probing pocket depth was measured using a periodontal probe at the mesiobuccal and mid-buccal sites for all present teeth except for the third molars. Prior to the start of this study, all examiners were trained and calibrated using a chart, periodontal models, and volunteers at the Nagasaki University Hospital. Tooth loss was defined as being in the lowest tertile for the number of remaining teeth (≤20 in men and ≤19 in women). In this study, periodontitis was defined as periodontal pocket depth ≥ 4.0 mm.

### 2.3. Statistical Analysis

We performed all statistical analyses using SAS for Windows (version 9.4; SAS Inc., Cary, NC, USA). Values of *p* < 0.05 were regarded as statistically significant.

The characteristics of the study population by functional atherosclerosis status were expressed as means ± SD for continuous valuables. They were expressed as *n* (%) for male sex, current smoker status, presence of decayed tooth, and periodontitis. Significant differences by functional atherosclerosis status were evaluated using Student’s *t*-test for continuous variables and the χ^2^ test for categorical variables.

Logistic regression was used to calculate odds ratios (ORs) and 95% confidence intervals (CIs) to determine the influence of CIMT on tooth loss. A trend test for tooth loss in relation to CIMT quartiles was also performed with logistic regression. Adjustments for confounding factors were made using two models. In the first model, adjustment was only made for sex and age. The second model included other possible confounding factors: systolic blood pressure (SBP) (mmHg), BMI (kg/m^2^), smoking status (never, former, or current smoker), γGTP (U/L), TG (mg/dL), HDLc (mg/dL), HbA1c (%), decayed tooth (yes or no), and periodontitis (yes or no). To evaluate the association between CIMT and tooth loss, we performed additional analyses stratified by functional atherosclerosis status. The *p*-value for the effect of the interaction between CIMT quartile and functional atherosclerosis status on tooth loss was also calculated using a multivariable model. Furthermore, simple correlation analysis of CIMT and CAVI based on functional atherosclerosis status was also conducted.

## 3. Results

### 3.1. Characteristics of the Study Population by Functional Atherosclerosis Status

The characteristics of study participants by functional atherosclerosis status are shown in Table 1. Compared to participants without functional atherosclerosis, a higher proportion of participants with functional atherosclerosis were men. They were also significantly older and had higher SBP, diastolic blood pressure (DBP), HbA1c, and CIMT. A higher proportion had periodontitis. Participants with functional atherosclerosis had significantly lower HDLc levels and fewer remaining teeth than participants without functional atherosclerosis.

### 3.2. Association between CIMT and Tooth Loss among All Study Participants

Table 2 shows the association between CIMT and tooth loss among all study participants. A significant positive association between CIMT (continuous value) and tooth loss was observed in the sex- and age-adjusted model. After further adjustment for known confounding factors, the association was not statistically significant.

### 3.3. Association between CIMT and Tooth Loss by Functional Atherosclerosis Status

Table 3 shows the association between CIMT and tooth loss stratified by functional atherosclerosis status. We observed a significant positive association between CIMT and tooth loss in participants without functional atherosclerosis, but not in participants with functional atherosclerosis. These associations were unchanged after further adjustment for known confounding factors.

### 3.4. Effect of The Relationship between CIMT and Functional Atherosclerosis on Tooth Loss

The interaction between CIMT and functional atherosclerosis had a significant effect on tooth loss (*p* = 0.015 in the sex- and age-adjusted model and *p* = 0.019 in the fully adjusted model).

### 3.5. Correlation between CIMT and CAVI by Functional Atherosclerosis Status

Simple correlation coefficients for CIMT and CAVI by functional atherosclerosis status are shown in Table 4. A significant correlation was observed between CIMT and CAVI in participants without functional atherosclerosis. No significant associations were observed in participants with functional atherosclerosis.

### 3.6. Sex-Specific Analysis of The Association between CIMT and Tooth Loss by Functional Atherosclero Sis Status

In sex-specific analyses, we found essentially the same associations for both men and women. Among participants without functional atherosclerosis, the fully adjusted OR (95% CI) for tooth loss and a 1 SD increment in CIMT was 1.49 (1.05–2.09) for men and 1.19 (0.92–1.54) for women. Among participants with functional atherosclerosis, the corresponding values were 0.98 (0.69–1.40) for men and 0.99 (0.68–1.44) for women.

## 4. Discussion

The major finding of the present study is that the positive association between CIMT and tooth loss was observed only in participants without functional atherosclerosis. A previous Japanese twin study with participants over the age of 50 years showed that the number of teeth was significantly related to structural atherosclerosis evaluated by CIMT [10]. This study is compatible with our present study because our study also shows a positive association between CIMT and tooth loss.

Our present study provides further evidence that the positive association between CIMT and tooth loss is observed only in participants without functional atherosclerosis. These associations were unchanged even after further adjustment for known confounding factors.

**Figure 1:**
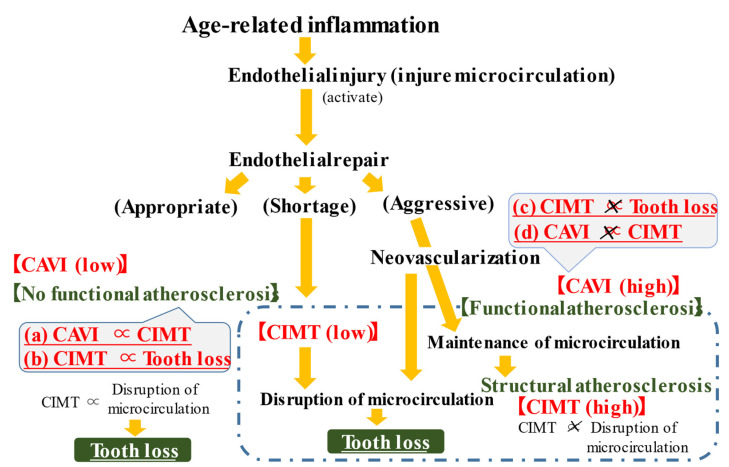
Possible mechanisms underlying the carotid intima-media thickness (CIMT) and tooth loss. Relations in red (a)~(d) were observed in this study. Increased CIMT reflects disruption of microcirculations among participants without functional atherosclerosis but not among those with functional atherosclerosis. CAVI: cardio-ankle vascular index, CIMT: carotid intima-media thickness.

A summary of the possible mechanism underlying the present results is shown in Figure 1. Only among participants without functional atherosclerosis, CIMT was revealed to be positively associated with tooth loss. Disruption of the microcirculation might have an important role in this association.

Values of functional arterial stiffness as evaluated with CAVI reflect the condition of the microcirculation [18]. Neovascularization, which plays an important role in the maintenance of the microcirculation [19], is also observed in atherosclerotic lesions [20,21]. Therefore, progression of atherosclerosis (structural arterial stiffness) has a partly beneficial role in maintaining the microcirculation. Disruption of the microcirculation results in common age-related diseases such as hypertension and reduced muscle strength [22,23]. Active progression of structural atherosclerosis might have a beneficial influence on maintaining muscle strength among elderly participants with hypertension [24,25], which is closely related to alterations in the microcirculation [26] and structural atherosclerosis [27]. In addition, a previous study with healthy elderly individuals reported that tooth loss is more strongly associated with masseter muscle thickness than with aging [28]. That study also reported that masseter muscle thickness is associated with grip strength. Therefore, these studies support the possible beneficial role of progressive structural atherosclerosis in maintaining the microcirculation and preventing tooth loss.

However, factors that stimulate the progression of structural atherosclerosis are harmful to the maintenance of the microcirculation. In fact, age-related low-grade inflammation is reported to be associated with atherosclerosis and disruption of the microcirculation [22,29]. Among participants without functional atherosclerosis, low CIMT values indicate a low risk of microcirculation disruption and high CIMT values indicate higher microcirculation maintenance activity (endothelial repair), which is stimulated by stronger endothelial damage. In such individuals, CIMT can act as an indicator of the condition of the microcirculation. Among participants with functional atherosclerosis, low CIMT values indicate a deficiency of the capacity to maintain the microcirculation and high CIMT values indicate more disruption of the microcirculation. Since the status of the microcirculation might be associated with oral health [30], CIMT can be associated with tooth loss only among participants without functional atherosclerosis [Table 3 and Figure 1b,c]. In the present study, a small but significant positive association between CIMT and CAVI was observed among participants without functional atherosclerosis [Table 4 and Figure 1a], but not among participants with functional atherosclerosis [Table 4 and Figure 1d]. These findings support the proposed mechanism.

Unlike other epidemiological studies, the strengths of our study are that we conduced multi-faceted analyses in a simple study population and all of our results could be explained by simple mechanisms.

One clinical implication of the present study is that progression of structural atherosclerosis is one aspect of aggressive endothelial repair, which is stimulated by endothelial injury. Progression of structural atherosclerosis (increased arterial stiffness) is generally regarded as a cardiovascular risk factor. However, when formulating a better strategy for treating or preventing diseases related to the microcirculation, including tooth loss, the beneficial influence of increased structural arterial stiffness should be considered. The present study revealed that a combination of CAVI and CIMT might be an efficient way to evaluate arterial stiffness.

Potential limitations of this study warrant consideration. The condition of the microcirculation is important to the present results, but no data to evaluate activity related to the maintenance of the microcirculation was available. Further investigations with markers of oxidative stress such as reactive oxygen species and antioxidant enzymes [31], markers of endothelial injury such as endocan and angiopoietin-2 [32], and markers of endothelial repair such as CD34-positive cell count [4,33] are necessary. Since this was a cross-sectional study, causal relationships could not be established.

## 5. Conclusions

In conclusion, independent of known confounding factors, CIMT is positively associated with tooth loss in participants without functional atherosclerosis but not in participants with functional atherosclerosis. These findings help clarify the influence of the progression of arterial stiffness on tooth loss.

## Figures and Tables

**Table 1:** Characteristics of the study population by functional atherosclerosis status.

	Functional Atherosclerosis		
	(−)	(+)	*p*
Number of participants	846	389	
Men, %	33.4	52.1	<0.001
Age, years	65.2 ± 10.0	74.8 ± 7.2	<0.001
SBP, mmHg	135 ± 19	144 ± 19	<0.001
DBP, mmHg	82 ± 11	83 ± 12	0.042
BMI, kg/m^2^	22.9 ± 3.5	23.2 ± 3.0	0.134
Current smoker, %	8.7	6.9	0.282
Serum γ-GTP, U/L	30 ± 31	30 ± 44	0.864
Serum TG, mg/dL	101 ± 61	104 ± 50	0.436
Serum HDLc, mg/dL	64 ± 15	59 ± 14	<0.001
HbA1c, %	5.7 ± 0.5	5.9 ± 0.6	<0.001
Participants with decayed teeth, %	59.1	63.2	0.637
Participants with Periodontitis, %	47.5	56.0	0.005
CIMT, mm	0.67 ± 0.13	0.74 ± 0.13	<0.001
Number of remaining teeth	22 ± 7	19 ± 9	<0.001

Values are means ± standard deviation unless otherwise indicated. SBP: systolic blood pressure, DBP: diastolic blood pressure, BMI: body mass index, γ-GTP: γ-glutamyltranspeptidase, TG: triglycerides, HDLc: high-density lipoprotein cholesterol. HbA1c: hemoglobin A1c, CIMT: carotid intima-media thickness.

**Table 2:** Odds ratios and 95% confidence intervals for tooth loss by carotid intima-media thickness (CIMT).

	Carotid Intima-Media Thickness (CIMT) Quartile				*p* for Trend (Quartile)	1 SD Increment in CIMT
	Q1 (Low)	Q2	Q3	Q4 (High)		
Total						
Number of participants	305	316	310	304		
Number of tooth loss (%)	55 (18.0)	90 (28.5)	120 (38.7)	141 (46.4)		
Sex- and age-adjusted OR	1.00	1.08 (0.72, 1.64)	1.24 (0.82, 1.87)	1.27 (0.83, 1.95)	0.215	1.16 (1.01, 1.34)
Multivariable OR	1.00	1.15 (0.76, 1.76)	1.23 (0.80, 1.88)	1.26 (0.81, 1.95)	0.312	1.14 (0.98, 1.32)

Tooth loss was defined as being in the lowest tertile for the number of remaining teeth (≤20 in men and ≤19 in women). Multivariable odds ratios (ORs) were adjusted for sex, age, systolic blood pressure (SBP), body mass index (BMI), smoking status, γ-glutamyltranspeptidase (γ-GTP), triglycerides (TG), high density lipoprotein cholesterol (HDLc), hemoglobin A1c (HbA1c), presence of decayed tooth, and presence of periodontitis. Median carotid intima-media thickness (CIMT) values for men were 0.56 mm in quartile 1, 0.66 mm in quartile 2, 0.75 mm in quartile 3, and 0.87 mm in quartile 4. For women, the corresponding values were 0.54 mm in quartile 1, 0.62 mm in quartile 2, 0.71 mm in quartile 3, and 0.83 mm in quartile 4. The 1 standard deviation (SD) increment in CIMT was 0.71 mm for men and 0.68 mm for women.

**Table 3:** Odds ratios and 95% confidence intervals for tooth loss in relation to carotid intima-media thickness (CIMT) by functional atherosclerosis status.

	Carotid Intima-Media Thickness (CIMT) Quartile				*p* for Trend (Quartile)	1 SD Increment in CIMT
	Q1 (Low)	Q2	Q3	Q4 (High)		
**Participants without functional atherosclerosis**						
Number of participants	257	235	196	158		
Number of tooth loss (%)	38 (14.8)	56 (23.8)	69 (35.2)	77 (48.7)		
Sex and age-adjusted OR	1.00	1.02 (0.62, 1.67)	1.31 (0.79, 2.17)	1.76 (1.04, 3.00)	0.017	1.29 (1.07, 1.55)
Multivariable OR	1.00	1.13 (0.68, 1.88)	1.40 (0.83, 2.36)	1.81 (1.05, 3.12)	0.021	1.27 (1.04, 1.55)
**Participants with functional atherosclerosis**						
Number of participants	48	81	114	146		
Number of tooth loss (%)	17 (35.4)	34 (42.0)	51 (44.7)	64 (43.8)		
Sex- and age-adjusted OR	1.00	1.15 (0.53, 2.51)	1.02 (0.48, 2.15)	0.80 (0.38, 1.68)	0.312	1.00 (0.79, 1.26)
Multivariable OR	1.00	1.16 (0.52, 2.60)	0.91 (0.42, 1.98)	0.73 (0.34, 1.58)	0.212	0.99 (0.77, 1.26)

Tooth loss was defined as being in the lowest tertile for the number of remaining teeth (≤20 in men and ≤19 in women). Functional atherosclerosis was defined as cardio-ankle vascular index (CAVI) ≥ 9.0. Multivariable odds ratios (ORs) were adjusted for sex, age, systolic blood pressure (SBP), body mass index (BMI), smoking status, γ-glutamyltranspeptidase (γ-GTP), triglycerides (TG), high density lipoprotein cholesterol (HDLc), hemoglobin A1c (HbA1c), presence of decayed tooth, and presence of periodontitis. Median carotid intima-media thickness (CIMT) values for men were 0.56 mm in quartile 1, 0.66 mm in quartile 2, 0.75 mm in quartile 3, and 0.87 mm in quartile 4. For women, the corresponding values were 0.54 mm in quartile 1, 0.62 mm in quartile 2, 0.71 mm in quartile 3, and 0.83 mm in quartile 4. The 1 standard deviation (SD) increment in CIMT was 0.71 mm for men and 0.68 mm for women.

**Table 4:** Simple correlation coefficients for carotid intima-media thickness (CIMT) and cardio-ankle vascular index (CAVI) by functional atherosclerosis status.

	Functional Atherosclerosis			
	(−)		(+)	
	r	*p*	r	*p*
	CAVI		CAVI	
Number of participants	846		389	
CIMT	0.27	<0.001	0.01	0.784

Functional atherosclerosis was defined as CAVI ≥ 9.0. r: simple correlation coefficient, CAVI: cardio-ankle vascular index, CIMT: carotid intima-media thickness.

## Data Availability

According to ethical guidelines in Japan, we cannot provide individual data due to participant privacy considerations. In addition, the informed consent obtained does not include a provision for publicly sharing data. Qualified researchers may apply to access a minimal dataset by contacting Prof. Takahiro Maeda, Principal Investigator, Department of General Medicine, Nagasaki University, Nagasaki, Japan at tamaeda@nagasaki-u.ac.jp or the Office of Data Management at ritouken@vc.fctv-net.jp. Information about data requests is also available online at: https://www.mh.nagasaki-u.ac.jp/soshin/ (accessed on 7 July 2022). and http://www.med.nagasaki-u.ac.jp/cm/ (accessed on 7 July 2022).
